# Comparison of Antimicrobial Susceptibility of *Campylobacter *Strains Isolated from Food Samples and Patients with Diarrhea 

**DOI:** 10.7508/ibj.2016.02.004

**Published:** 2016-04

**Authors:** Bita Bakhshi, Amin Naseri, Masoud Alebouyeh

**Affiliations:** 1 Dept. of Bacteriology, Faculty of Medical Sciences, Tarbiat Modares University, Tehran, Iran;; 2 Research and Science Branch, Islamic Azad University, Tehran, Iran;; 3 Foodborne and Waterborne Diseases Research Center, Research Institute for Gastroenterology and Liver Diseases, Shahid Beheshti University of Medical Sciences, Tehran, Iran

**Keywords:** *Campylobacter*, Genetic diversity, Drug resistance

## Abstract

**Background:**

*Campylobacter* infections may lead to serious conditions, including septicemia or other invasive forms of the disease, which require rapid and accurate laboratory diagnosis and subsequently appropriate antimicrobial therapy. The aim of this study was to compare the species distribution and antimicrobial susceptibility pattern of *Campylobacter* spp. strains isolated from patients and food samples.

**Methods:**

Biochemical identification was performed on 15 clinical and 30 food isolates of *Campylobacter* recovered onto Brucella agar containing 5% sheep blood. PCR was carried out to confirm the identity of *Campylobacter* spp. using primers for *cadF*, *hipO*, and *asp* genes of *Campylobacter*. To determine antibiotic sensitivity of isolates, Kirby-Bauer assay was carried out using 16 different antibiotic discs.

**Results:**

PCR assay and biochemical tests confirmed all 45 isolates as *Campylobacter*: 20 (44.44%) as *C. jujeni*, 10 (22.22%) as *C. coli*, and 15 (33.34%) as other *Campylobacter* strains. The maximum resistance was observed to cefotaxime and imipenem (each 86.49%) and the maximum sensitivity to erythromycin (48.65%).

**Conclusion:**

*C. jujeni* is dominant among isolates from clinical and food samples. In addition, tetracycline remains the first-line therapeutic agent against *Campylobacter* infections in Iran.

## INTRODUCTION

*Campylobacter* spp. are motile, oxidase-positive microorganisms with spiral or corkscrew appearance belonging to a group of Gram-negative microaerophilic bacteria^[^^1^^]^. Many *Campylobacter* spp. have been reported to be implicated in human diseases, such as Campylo-bacteriosis, periodontitis, diarrhea etc.^[^^2^^,^^3^^]^. However, *C. jejuni* and *C. coli* are the most common isolates from human pathological samples, including enteritis^[^^1^^,^^4^^]^. In addition, *C. fetus* is also seen as an opportunistic pathogen in human^[^^5^^]^.

Most estimates of incidence in developing countries are from laboratory-based surveillance of pathogens responsible for diarrhea. Isolation rates of* Campylo-bacter *in developing countries range from 5 to 20%^[^^6^^,^^7^^]^. Although in most of the cases *Campylobacter *infections are self-limiting, some serious conditions may happen, such as diarrhea, cramping, abdominal pain, and fever within two to five days after exposure to the organism. Diarrhea may be bloody and can be accompanied by nausea and vomiting. In persons with compromised immune systems, Campylobacter occasionally spreads to the bloodstream and causes a serious life-threatening infection^[^^6^^]^. Some people develop arthritis and others may develop a rare disease called Guillain-Barré syndrome that affects nerves of the body and begins several weeks after the diarrheal illness^[^^8^^]^. Accordingly, rapid and accurate identification of the causing strains and the selection of the most efficient therapeutics are required^[^^9^^]^. However, increasing antimicrobial resistance and different patterns of antibiotic susceptibility among different clinical and environmental isolates of *Campylobacter* have been frequently reported by some investigations^[^^10^^,^^11^^]^. For example, in the late 1980s, resistance to quinolones was increased in Asia and Europe, following the introduction and indiscriminate use of these drugs in livestock^[^^12^^]^. Interestingly, despite the widespread use of erythromycin, resistance of *Campylobacter* to this antibiotic has remained low in industrialized countries^[^^8^^]^.

Biochemical and molecular methods are used for identification of *Campylobacter* spp. and strains. Many methods used to identify *Campylobacter* spp. are based on classic phenotypes of these bacteria, e.g. morphology, growth temperature, biochemical and serological reactions, and tolerance to higher temperatures^[^^13^^-^^15^^]^. Moreover, commercial kits such as enzyme immunoassay and ProSpecT Campylobacter Microplate Assay are available for direct and rapid identification of antigens from *C. jejuni* and *C. coli *in stool^[^^16^^,^^17^^]^. The aim of this study was to characterize *Campylobacter* spp. strains isolated from food and clinical samples using biochemical methods and PCR assay and to determine their antibiotic susceptibility patterns by disc diffusion test.

## MATERIALS AND METHODS

**Bacterial strains**

The current study included 15 clinical and 30 food *Campylobacter *spp. isolates that were obtained during a 14-month period (June 2004- July 2005).The food isolates were collected from different areas of Tehran, 

Iran, including shopping centers and retails. Clinical isolates were obtained from patients referred to one major hospital in Tehran. The isolates were stocked in skim milk medium containing 15% glycerol and preserved at -20°C until use.

**Growth conditions and biochemical tests**

Campylobacter strains were cultivated on Brucella agar containing 5% sheep blood, vancomycin, polymyxin B and trimethoprim (pH 7.2±0.2) at 42°C for 48 h. The cultivation was performed under microaerobic conditions provided by gas replacement method^[^^18^^]^. Gram staining, catalase and urease production, nitrate reduction^[^^19^^]^, hippurate hydro-lysis^[^^20^^]^, and indoxyl acetate hydrolysis^[^^21^^]^ tests were used for biochemical identification and bio-typing of the strains.

**Molecular characterization by PCR**

PCR was carried out to detect *Campylobacter* species^[^^22^^]^. DNA was extracted using boiling method. Briefly, a clone of the bacteria was suspended in 200 μl sterile distilled water, boiled for 10 min and incubated at -20°C for 10 min. It was then centrifuged at 24148 ×g at room temperature for 10 min and the supernatant was used as template DNA in PCR reaction.

PCR amplification was performed using primers specific for different genes, *Campylobacter* adhesion genes: fibronectin gene (*cadF*) for detection of *Campylobacter* genus, hippuricase or benzoylglycine amidohydrolase gene (*hipO*) for *C. jejuni*, and aspartokinase or aspartate kinase gene (*asp*) for *C. coli* (Table 1). For PCR, 0.3 μl dNTP (25 mM), 0.2 μl Taq polymerase (5 unit/μl), 0.6 μl MgCl_2_ (50 mM), 5 μl DNA template, 0.25 μl primer-F (100 pM), 0.25 μl primer-R (100 pM), and 2.5 μl PCR buffer (10×) were mixed and brought to a volume of 25 μl using distilled water.

The final volume was subjected to PCR amplification within a thermal cycler (Eppendorf, Germany) with the initial denaturation at 95°C for 5 min (for all three genes), followed by 30 amplification cycles. The amplification cycles included denaturation at 95°C for 45 seconds (for all three genes), annealing at 43°C (*cadF*), 55°C (*hipO*) and 52°C (*asp*) for 1 min, extension at 72°C for 1 min (for all three genes), and the final extension at 72°C for 5 min.

**Table 1:** Characteristics of the primers used for detection of *Campylobacter *isolates by PCR

**Gene**	**Primer**	**Sequence**	**Length (nt)**	**Band (bp)**
*cadF*	Forward Reverse	5'-TTGAAGGTAATTTAGATATG-3' 5'-CTAATACCTAAAGTTGAAAC-3'	20 20	400
				
*hipO*	Forward Reverse	5'-GAAGAGGGTTTGGGTGGTG-3' 5'-AGCTAGCTTCGCATAATAACTTG-3'	19 23	735
				
*asp*	Forward Reverse	5'-GGTATGATTTCTACAAAGCGAG-3' 5'-ATAAAAGACTATCGTCGCGTG-3'	22 21	500

bp, base pair; nt, nucleotide

Afterwards, 8 μl PCR amplicons together with 2 μl loading buffer were run on 1% agarose gel in 1× Tris-Borate-EDTA buffer at 95 V for 2 h. Finally, the electrophoresis gel was stained in ethidium bromide solution (10 μg/ml), and visualized at wavelength of 590 nm under an ultraviolet trans-illuminator (Medox Biotech, India).

**Antibiotic susceptibility test**

Antibiotic susceptibility assay was performed using Kirby-Bauer disk diffusion test^[^^23^^]^. For this purpose, 16 antibiotic disks were chosen (μg/disc), including erythromycin (15), azithromycin (15), gentamicin (10), ampicillin (30), tetracycline (5), imipenem (10), ciprofloxacin (5), nalidixic acid (30), kanamycin (5), chloramphenicol (30 μg/disc), streptomycin (30), cefotaxime (30), trimethoprim (30), cefepime (30), tobramycin (10), and amikacin (30), which all were purchased from MAST Company, UK. The susceptibility of the bacteria to each antibiotic was determined according to the latest guidelines published by the Clinical and Laboratory Standards Institute.

## RESULTS

**Biochemical identification of isolates**

Results of Gram staining, nitrate reduction, catalase and urease production, and indoxyl acetate hydrolysis tests revealed that all 45 suspected isolates belonged to

*Campylobacter* spp. Among them, the hippurate hydrolysis test identified 20 isolates (44.44%) as *C. jujeni* and 10 isolates (22.22%) as *C. coli*. The remaining 15 (33.34%) *Campylobacter* spp. isolates belonged to other species.

**Molecular characterization by PCR**

**Fig. 1:**
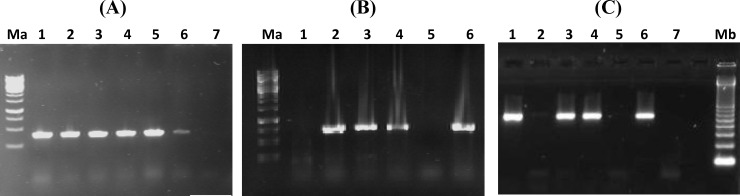
(A) PCR amplification of *cadF* (400 bp): Lane1, positive control (*C. jejuni* ATCC 29428), lanes 2-6, patient or food samples with positive results; lane7, negative control. (B) PCR amplification of *hipO* (735 bp): Lane1, negative control, lane 2, positive control (*C. jejuni* ATCC 29428), lanes 3-6, patient or food samples. (C) PCR amplification of *asp* (500 bp): lane1, positive control (*C. coli* ATCC 43478); lanes 2-6, patient or food samples; lane 7, negative control. Ma, 1 kb DNA ladder (Fermentas, USA) and Mb, 100 bp DNA ladder (Fermentas, USA). Negative control is DNA from *Escherichia coli* ATCC 25922

The result of PCR assay confirmed the biochemical test results for detection of *Campylobacter* spp. and related species. Using *cadF* primers, all isolates revealed a 400-bp band on electrophoresis, which indicated the presences of *Campylobacter* spp. (Fig. 1A). Amplification with* hipO* primers revealed 20 *C. jujeni *among the isolates (Fig. 1B). In addition, a 500-bp band of *asp* gene was detected in 10 isolates corresponding to the presence of *C. coli* (Fig. 1C).

**Antibiotic sensitivity test**

Antibiotic sensitivity testing was performed on clinical and food isolates of *Campylobacter *spp. Disregarding the source of isolates, maximum resistance was observed to cefotaxime and imipenem, each seen among 86.49% of the isolates. In addition, the maximum sensitivity belonged to erythromycin, observed among 48.65% of the isolates. Among the strains isolated from food samples, the maximum resistance was seen to imipenem (90.9%), and the maximum sensitivity to chloramphenicol (40.9%). Moreover, among the isolates from clinical samples, the maximum resistance belonged to cefotaxime and trimethoprim (each, 93.34%), and the maximum sensitivity to tobramycin (46.66%). Comparisons of the susceptibility data among *Campylobacter* strains isolated from clinical and food samples were performed by SPSS software (version 19), and no significant difference was observed between the two groups (*P*>0.05). 

## DISCUSSION

Due to the problems associated with the application of biochemical and biological methods for identification of *Campylobacter* strains, there are overwhelming intricacies about characterization and epidemiological studies of these bacteria. In the present study, we used both biochemical and molecular methods for detection of *Campylobacter* isolates from food and clinical samples. Although our results revealed no difference between the sensitivity of PCR assay and that of biochemical tests for species identification of *Campylobacter* isolates, many advantages have been suggested for molecular approaches in making differentiation between various strains of the bacteria. First, the accuracy and speed of molecular diagnostics are higher than biochemical methods with no need to long incubation at different temperatures. Second, molecular techniques are easier and less costly than biochemical methods, and they are more suitable for epidemiological studies.

Using PCR assay, we found that among 45 studied isolates, almost 45% were *C. jujeni* and 23% *C. coli*, and the remaining belonged to other *Campylobacter* species. Given the clinical significance of *C. jujeni* and *C. coli*, our overall focus was on these two species. These findings are in accordance with previous studies that have shown the dominance of *C. jujeni* over *C. coli*^[^^24^^-^^26^^]^. For instance, Denis *et al.*^[^^24^^]. ^observed that among 513 isolates from chickens, 61.5% were *C. jujeni* and 38.5%* C. coli*. In addition, Fitzgerald *et al.*^[^^25^^] ^in an investigation on isolates from farm and clinical environments suggested that the higher frequency of *C. jujeni* over the *C. coli* may be due to the extensive colonization of the *C. jujeni* in a vast range of hosts living as commensalism. Moreover, Manfreda *et al.*^[^^27^^] ^found that *C. jujeni* is dominant among isolates in cold seasons, while *C. coli* is more frequent in warm seasons as a thermo-tolerant species; however, in the current study, the overall dominance is attributed to the *C. jujeni*, regardless of its seasonality.

In the present study, disc diffusion test was performed to determine the resistance pattern of isolates to antibiotics belonging to aminoglycosides, quinolones, beta-lactams, sulfonamides, and cephalo-sporins family of antibiotics according to the globally accepted standard criteria. We observed that the maximum resistance belonged to cefotaxime and imipenem and then to nalidixic acid, trimethoprim, ampicillin, ciprofloxacin, tetracycline, streptomycin, and kanamycin, respectively. We also found that the maximum sensitivity was to erythromycin and then to chloramphenicol, gentamicin, azithromycin, and cefepime, respectively. Senok and colleagues^[^^28^^] ^also indicated a high degree of erythromycin sensitivity and ciprofloxacin resistance among *C. jejuni* isolates of human and poultry origin. Our study shows a high resistance to nalidixic acid and ciprofloxacin and low resistance to azithromycin and gentamicin. Antibacterial susceptibility test by Lehtopolku and coworkers^[^^29^^] ^on 1808 isolates isolated between 2003-2005 also showed high resistance to nalidixic acid (41.4% *C. jejuni* and 83.3 *C. coli* strains) and ciprofloxacin (42.4% *C*. *jujeni* and 83.3 *C. coli* strains) as well as low resistance to azithromycin (5% *C. jejuni* and 38.9 *C. coli* strains) and gentamicin (0.9% *C. jejuni* and 0 *C. coli* strains). In addition, Dadi and Asrat^[^^30^^]^ found the maximum susceptibility to erythromycin, chloramphenicol, amoxicillin, and the maximum resistance to ampicillin, gentamicin, tetracycline, streptomycin and kanamycin. Our findings are consistent with those of Dadi and Asrat^[^^30^^]^ with the exception of higher susceptibility to gentamicin that was observed in our study.

Moreover, Oza and colleagues^[^^31^^]^ reported the lowest resistance to ciprofloxacin (3%), which is in contrary with our findings. High resistance to ciprofloxacin in the present study may be due to the fact that fluoroquinolones such as ciprofloxacin are frequently used for treatment of campylobacteriosis because of their broad spectrum of activity against enteric pathogens^[^^32^^]^. Furthermore, Oza and colleagues^[^^31^^] ^observed the susceptibility to erythromycin, gentamicin, and chloramphenicol in more than 99% of human or poultry isolates of *Campylobacter* spp., whereas maximum antimicrobial resistance was seen for ampicillin, nalidixic acid and tetracycline, respectively. Thus, our findings together with the previous reports support the continued use of erythromycin and chloramphenicol as first-line therapy for enteritis of *Campylobacter* spp. in Iran and other countries encountering campylobacteriosis.

In the present investigation, the maximum resistance was observed to cefotaxime and imipenem (each 86.49%). In an earlier study by Tajada and colleagues^[^^33^^]^, all strains were susceptible to imipenem. Likewise, in another study among clinical *C. coli* and *C. jejuni* strains, imipenem was highly effective against multidrug resistance *campylobacter*^[^^29^^]^. Also, in a study in Kuwait during 2002-2010, 97 *C. jejuni* isolates were investigated, and no resistance to imipenem was observed^[^^34^^]^. High resistance to imipenem, which was observed among our isolates, makes further observation and tracking necessary for finding the exact molecular basis of such high resistance.

Tetracycline resistance was high among our isolates (78.38%). Albert^[^^34^^] ^and Gallay *et al.*^[^^35^^]^ showed that resistance to tetracycline has a tendency to increase from 2003-2010 and 1999-2004, respectively. The high rate of tetracycline resistance may be due to *tetO*, which is a plasmid encoding gene introduced to be responsible for tetracycline resistance in *Campylo-bacter* spp.^[^^36^^]^. 

In conclusion, we found that the isolation frequency of *C. jujeni* in both clinical and food specimens is higher than that of *C. coli*. Our findings indicated that the *Campylobacter* has the highest resistance to ciprofloxacin, nalidixic acid, and tetracycline as well as the least resistance to erythromycin and chlor-amphenicol, which still suggest the two latter as being first-line therapeutic agents against *Campylobacter* infections in Iranian clinics.
